# Proceedings of Veterinary Societies

**Published:** 1889-01

**Authors:** 


### PROCEEDINGS OF VETERINARY SOCIETIES.

OFFICERS UNITED STATES VETERINARY MEDICAL ASSOCIATION.—Semi-annual meeting, March 18, 1889, at Boston. Annual meeting, September 16, 1889.

*President*—Rush S. Huidekoper, 36th and Pine Streets, Philadelphia, Pa.

*Vice-President*—J. C. Myers, Jr., 379 Walnut street, Cincinnati, Ohio.

*Secretary*—W. H. Hoskins, 12 South 37th street, Philadelphia, Pa.

*Treasurer*—J. L. Robertson, 409 9th Avenue, New York.

*Board of Censors*—Dr. William L. Zuill, Pa.; Dr. E. C. Ross, Conn; Dr. J. F. Mechener, Mass.; Dr. W. H. Wray, N. Y.; Dr. L. H. Howard, Mass.; Dr. A. W. Clement, Md.; Dr. L. McLean, N. Y.

*Library Committee*—S. K. Johnson, Chairman, 117 West 25th street, N. Y.; William Dougherty, Md.

*Committee on Intelligence and Education*—A. W. Coates, Chairman, 141 West 54th street, N. Y.; J. C. Myers, Sr., Ohio; B. McInnes, S. C.; A. Peters, Mass.; W. L. Zuill, Pa.

*Finance Committee*—L. McLean, Chairman, 14 Nevins street, Brooklyn, N. Y.; William Bryden, Mass.; C. B. Mechener, N. Y.

*Committee on Diseases*—A. W. Clement, Chairman, 210 St. Paul street, Baltimore, Md.; J. F. Winchester, Mass.; W. E. B. Miller, N. J.; R. A. McLean, N. Y., Paul Paquin, Mo.

*Prize Committee*—L. H. Howard, Chairman, 1440 Washington street, Boston, Mass.; Dr. J. Dixon, N. J.; J. Gerth, Jr., N. J.

*Special Committee*—To secure a uniform standard of examinations by the different veterinary colleges of North America—M. Bunker, Chairman, Newton, Mass.; C. C. McLean, Pa.; S. J. J. Harger, Pa.

*Committee on Army Legislation*—A. Liautard, Chairman, 141 West 54th street, N. Y.; W. H. Pendry, Brooklyn, N. Y.; C. Moulton, Mo.

*Publication Committee*—W. H. Hoskins, Chairman, 12 South 37th street, Philadelphia, Pa.; C. B. Mechener, N. Y.; D. J. Dixon, N. J.

*Assistant State Secretaries*—P. Z. Colsson, V. S., Mobile, Ala.; Ward B. Rowland, D. V. S., Los Angeles, Cal.; F. W. McClellan, V. S., 144 Noble street, Bridgeport, Conn.; Joseph C. Bushman, V. S., 404 14th street, N. W. Washington, D. C.; Madison Bunker, D. V. S., Newton, Mass.; George H. Bailey, D. V. S., 1 Pine street, Portland, Me.; William Dougherty, D. V. S., 1035 Cathedral street, Baltimore, Md.; C. C. Lyford, V. S., Minneapolis, Minn.; C. W. Crowley, D. V. S., St. Louis, Mo.; H. J. Detmars, V. S., Champaign, Ill.; D. J. Dixon, D. V. S., 366 Washington street, Hoboken, N. J.; J. Faust, V. S., 211 Main street, Poughkeepsie, N. Y.; William R. Stowe, V. S., Dayton, Ohio; Charles T. Gœntner, D. V. S., Bryn Mawr, Pa.;

Charles H. Peabody, D. V. S., Main and Worcester streets, Providence, R. I.; B. McInnes, V. S., Charleston, S. C.; E. W. Rowland, D. V. S., Monroe, Wis.; J. W. Scheibler, D. V. S., 321 2d st., Memphis, Tenn.

New York State Academy of Veterinary Science and Comparative Pathology.—At the November and December meetings of this Society the subject of roaring in horses elicited quite an amount of discussion among its members, owing to a paper on this subject which was read by Dr. Robert Ward, M. R. C. V. S., of Baltimore, Md. The discussion was opened by Dr. James Hamill saying that if Dr. Fleming claimed success in operating for this disease in horses, there must be some good ground for its success, as he (Fleming) is the one man of all men alive who has done the most for the veterinary profession in a scientific manner. Dr. Hamill also claimed that there were several conditions causing roaring, some of which could be removed by operation, and that he would not hesitate for a moment to perform such an operation.

Dr. H. D. Gill agreed with the above, claiming that it was absolutely necessary to diagnose the cause *correctly*, that some of the causes can be removed without doubt, and permanent relief obtained.

Dr. L. V. Plageman, M. R. C. V. S., claimed that the success of the operation had not been such as would establish proof of a permanent cure, but, as veterinary science was advancing, he hoped that we would be enlightened on this subject in the near future.

Dr. Dancer, of Orange, N. J., described a special tube for larynx which, after a fair trial, gave no better results than the old tracheotomy tube.

D. H. E. Earl claimed that where paralysis was the cause it would be progressive and would yield to a certain extent to galvanism. He also censured the veterinarians for being to conservative in operating.

Dr. E. J. Breder admitted that an animal might be relieved, but never permanently cured.

Dr. P. Peters gave a history of all the operations and in not one case was there a single cure. Dr. P. had no confidence in the operation with our present knowledge.

Dr. J. M. Heard, M. R. C. V. S., thought that the result of the operation would be as liable to diminish as to increase the calibre of the air passage, but admitted from some of the illustrations exhibited, that there was a possibility of one or two conditions being removed from which relief could be obtained.

During the discussion natural wet and dry, and one artificial specimen of larynx and trachea were exhibited. The anatomy of the effected part was explained and a fullest confidence expressed by exhibitor as to the success of the operation, which will be published when circumstances warrant.

PENNSYLVANIA STATE VETERINARY MEDICAL ASSOCIATION.—A special meeting of the Association was held at the Veterinary Department, University of Pennsylvania, on Thursday, December 13. The meeting was called to order by the President, Dr. Thomas B. Raynor, at 2.30 P. M.

The President stated the object of the meeting to be the consideration of the ways and means for the presentation and passage of an act in the Pennsylvania Legislature regulating the practice of veterinary medicine in the Commonwealth. Dr. Hoskins interrogated the chair as to the extent of the duties of the committee appointed one year ago, and recommended that the committee of the Pennsylvania and Keystone Associations act in conjunction. Discussion in regard to amount of money to be appropriated for expenses of committee followed. Dr. Kooker read a list of veterinarians who could be depended upon to obtain the good services of the representatives of their own and adjoining counties. Letters of encouragement were read from Drs. Joentner, McLean, Collins, Jesse Baker, Esq., and others.

The sum of $100 was appropriated toward the expenses of the committee. The further sum of $300 was guaranteed, if necessary, toward the same purpose.

The following is the proposed form of bill :—

AN ACT TO REGULATE THE PRACTICE OF VETERINARY MEDICINE AND SURGERY IN PENNSYLVANIA.

SECT. 1. *Be it Enacted by the Senate and House of Representatives of the Commonwealth of Pennsylvania, in General Assembly met, and it is hereby enacted by authority of the same*, That every person who shall assume, or use, or cause to be used, any title pertaining to the practice of Veterinary Medicine or Surgery, or any of the branches of Veterinary Medicine or Surgery, shall be a graduate of a legally-chartered Veterinary College or University, having the power or authority to confer the degree of Veterinary Surgeon or analogous title ; except as provided for in SECT. 2. And such practitioner shall be required to register in the book kept for that purpose, in the office of the Prothonotary of the county in which he resides.

SECT. 2. Any person who has assumed the title of Veterinary Surgeon or analogous title, in this Commonwealth, for the five years preceding the passage of this Act, without being entitled to the degree of Veterinary Surgeon or analogous title, shall be allowed to continue the use of the title ; but such person shall appear before the Prothonotary of the county in which he resides and make affidavit of that fact ; he shall then be recorded as an "existing practitioner."

SECT. 3. The Prothonotary shall purchase a book of suitable size, to be known as the *Veterinary Medical Register* of the county, and shall set apart one full page for the registration of each practitioner ; and, when any practitioner shall die or remove from the county, the Prothonotary shall make a note of the same, and shall perform such other duties as are required by this Act.

SECT. 4. Every practitioner who shall be admitted to register shall pay to the Prothonotary the sum of one dollar ; which sum shall be a compensation in full for registration. The Prothonotary shall give a receipt for the same, and such registration shall take place within six months from the passage of this Act.

SECT. 5. Nothing in this Act shall be so construed as to prevent any Veterinary Surgeon (if legally qualified to use the title) from using the title of "Veterinary Surgeon" or analogous title in this Commonwealth; but if such Veterinary Surgeon opens an office or uses the title for the transaction of business, he shall be deemed "a sojourner" and shall conform to the requirements of this Act.

SEC. 6. Any person who may desire to commence the practice of Veterinary Surgery or Medicine, or any of its branches, in this State, after the passage of this Act, and who holds a Veterinary diploma, issued, or purporting to have been issued, by any Veterinary College or University in this State, another State or foreign country, shall make affidavit before the Prothonotary that his diploma has been regularly issued by a legally chartered Veterinary College or University, after which such person will be allowed to register as provided for in this Act.

SECT. 7. Any person who shall present to a Prothonotary a Veterinary diploma which has been obtained fraudulently; or which is, in whole or in part, a forgery; or shall make affidavit to any false statement, intended to be filed or registered; or shall use the title of Veterinary Sugeon or analogous title, without conforming to the requirements of this Act; or shall otherwise violate or neglect to comply with any of the provisions of this Act, shall be deemed guilty of a misdemeanor, and, on conviction, shall be punished for each and every offence, by a fine of one hundred dollars—one-half to be paid to the prosecutor and the other half to be paid to the county; or shall be imprisoned in the county jail of the proper county for a term not exceeding one year, or both or either, at the discretion of the Court.

---

THE JERSEY STATE VETERINARY SOCIETY.—The regular meeting of this Society was held on Thursday, November 1st, 1885, at Taylor's Hotel, Jersey Gity.

Dr. Joseph Nayler occupied the chair on account of the President Dr. J. C. Corlies not being present, and the meeting was called to order at 3 P. M., the regular order of business was proceeded with and Dr. Albert H. McIntosh of Morristown, and Dr. Paul Will of Jersey City, were elected as members, as both are graduates from regular veterinary colleges.

Dr. Wm. Herbert Lowe of Patterson, read a lengthy paper on "The Hypodermic and Intratrachical Methods of Exhibiting Remedies in Equine Practice" after which a very lively discussion took place, in which all members present took an active part.

Dr. Joseph Autenreith promised to read a paper at the next meeting which is to be held at Paterson, February 7th, 1889. His paper will be on an interesting subject to all practicing veterinary medicine and surgery, and a lively debate is expected to take place.

CHARLES KUEHNE, D. V. S.,

*Secretary.*

